# Transoral Styloidectomy for Eagle Syndrome: A Case Report With Intraoperative Footage

**DOI:** 10.7759/cureus.90100

**Published:** 2025-08-14

**Authors:** Marcin Masalski, Jakub Kurasz, Aleksandra Kosiorowska, Szczepan Barnaś, Krzysztof Morawski

**Affiliations:** 1 Institute of Medical Sciences, University of Opole, Opole, POL; 2 Department of Otorhinolaryngology, University Clinical Hospital of Opole, Opole, POL; 3 Student Research Group in Otorhinolaryngology, Institute of Medical Sciences, University of Opole, Opole, POL; 4 Department of Otolaryngology, Head and Neck Surgery, Wroclaw University of Science and Technology, Wrocław, POL

**Keywords:** eagle syndrome, intraoral styloidectomy, styloidectomy, surgical technique guide, video documentation

## Abstract

Eagle syndrome is a rare condition resulting from an elongated styloid process or calcified stylohyoid ligament compressing adjacent anatomical structures. The condition typically manifests with unilateral neck pain, facial discomfort, odynophagia, and, in some cases, neurological disturbances. Among the available treatment options, transoral styloidectomy is one of the two primary surgical approaches. Despite its technical simplicity and clinical advantages, this approach is infrequently utilized due to the rarity of this syndrome and limited surgical training materials. This technical report presents a detailed account of the transoral approach to styloidectomy, performed in conjunction with tonsillectomy, and supported by intraoperative video documentation. The described case involved a 64-year-old female diagnosed with bilateral elongated styloid processes (4.15 cm on the left and 4.00 cm on the right), who underwent transoral styloidectomy combined with tonsillectomy. The visual documentation presented in this report outlines the essential technical steps of the procedure and serves as an effective educational tool, facilitating the training of surgeons in this relatively straightforward but rarely performed procedure.

## Introduction

Eagle syndrome is a group of symptoms resulting from compression caused by the elongated styloid process on surrounding anatomical structures or from calcification of the stylohyoid ligament, connecting the styloid process to the hyoid bone ​[1]​. This condition was first described by the English physician Watt Weems Eagle in 1937. It has been observed to be more prevalent in females and tends to affect people over the age of 30 ​[2,3]​. The incidence of Eagle syndrome remains a subject of debate. An elongated styloid process is diagnosed when its length exceeds, depending on the source, 2.5 to 4.5 cm ​[1,2,4,5]​ and is present in about 4%-7% of the population; however, clinical symptoms are present in a small percentage of patients with an elongated process ​[1,2,6]​. 

A commonly reported clinical manifestation of Eagle syndrome is unilateral neck and facial pain, typically occurring on the side of the elongated styloid process. This pain may radiate to the mandible and the auricular region. Additionally, patients may experience a persistent sensation of a foreign body, odynophagia (painful swallowing), and changes in the tone of voice ​[7]​. The vascular variant of Eagle syndrome is less common and is characterized by nerve compression and impaired blood flow in the internal jugular vein or the internal and external carotid arteries. Presenting symptoms may include neurological symptoms such as facial hemiparesis (weakness of facial muscles on one side), speech disturbance, diplopia (double vision), blurred vision, tinnitus (ringing in the ear), or even hearing decline. This subtype involves direct vascular compression by the elongated styloid process, which can compromise cerebral circulation ​[7,8]​. 

The primary treatment for Eagle syndrome is surgery to shorten the elongated styloid process, known as styloidectomy. Given the low prevalence of Eagle syndrome, styloidectomy is not routinely performed in many medical centers, which can result in limited surgical exposure and expertise [1,9]. In addition, limited indications for dissection in the region of the styloid process significantly hinder the development of procedural proficiency. This lack of familiarity may contribute to diagnostic delays and suboptimal surgical planning. The choice of surgical approach, transoral or transcervical, is primarily determined by the experience and preference of the operator. The transoral approach accesses the styloid process through the mouth, providing a less invasive surgical pathway but is associated with a restricted surgical field. In comparison, the transcervical approach involves a horizontal or oblique incision along the anterior border of the sternocleidomastoid muscle, which may lead to more noticeable scarring and extended postoperative recovery. Therefore, it is important to increase the availability of specialized training materials on styloidectomy techniques to increase diagnostic and therapeutic awareness among physicians, resulting in faster and more effective treatment for patients with Eagle syndrome. 

Previous studies have described the surgical treatment of Eagle syndrome using both transoral [6,10,11] and transcervical [4,12,13] approaches, as well as comparative analyses between the two methods [1,14-16]. While certain publications provide illustrative intraoperative photographs ​[4,6,10,11,12,14]​, peer-reviewed articles featuring video documentation remain scarce. The aim of this study is to present a case involving the use of a transoral approach to the styloid process and to provide a detailed discussion of the styloidectomy procedure combined with tonsillectomy performed via this method, supplemented with intraoperative video material.

## Technical report

**Figure 1:**
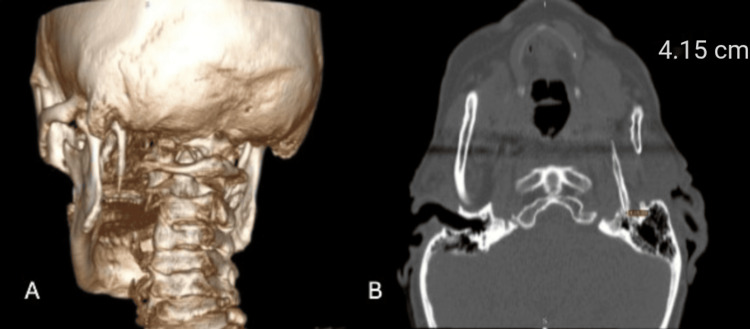
Volumetric craniofacial CT reconstruction showing the left styloid process (A). Measurement of the length of the styloid process (B).

A 64-year-old woman presented to an ENT outpatient clinic at Orthos Hospital, Komorowice near Wroclaw, Poland, with complaints of paroxysmal pain radiating to the mandible, neck, and both tonsillar regions, with symptoms more pronounced on the left side. She reported severe stabbing pain during swallowing, affecting both sides of the throat but predominantly localized to the left. The patient also reported tinnitus and a sensation of pressure in the left ear. Her medical history was notable only for cholelithiasis, with no relevant familial history. Physical examination of the oral cavity revealed palpable bilaterally elongated styloid processes in the region of the palatine tonsils; palpation of this area caused the patient considerable pain. A craniofacial CT scan showed bilateral elongated styloid processes, measuring 4.15 cm on the left side and 4.00 cm on the right side (Figure 1). A decision was made to proceed with a bilateral transoral styloidectomy combined with tonsillectomy. The transoral approach was selected due to the ease of access to the styloid process, which was palpable on the lateral pharyngeal wall. Additionally, the patient had no anatomical contraindications, such as a bulky tongue base, short neck, limited mouth opening, or restricted neck extension. The procedure was performed at Orthos Hospital by the first author of the article (M.M.). 

**Figure 2:**
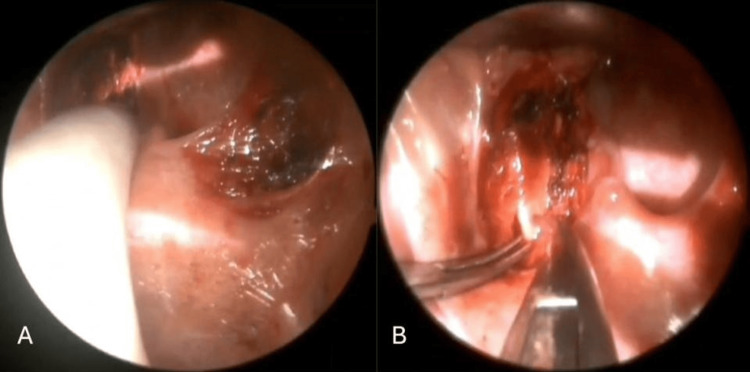
Palpation-based assessment of the tonsillar fossa and styloid process (A). Intraoperative exposure of the styloid process (B).

The surgical procedure was performed under general anesthesia using a head-mounted surgical light integrated with ocular magnifiers providing 3.0× magnification. The perioperative antibiotic prophylaxis consisted of cefazolin administration. After induction of general anesthesia, the patient was positioned supine with maximal neck extension. This positioning optimized surgical exposure of the palatine tonsillar region and facilitated approximation of the styloid process to the lateral pharyngeal wall. Following the insertion of a McIvor mouth gag, the palatoglossal and palatopharyngeal arches were injected with local anesthetic. Subsequently, incisions were made in both arches and the palatine tonsil was dissected and removed. Inspection of the tonsillar fossa revealed an elongated styloid process, which was identified and confirmed via palpation (Figure 2A).

**Figure 3:**
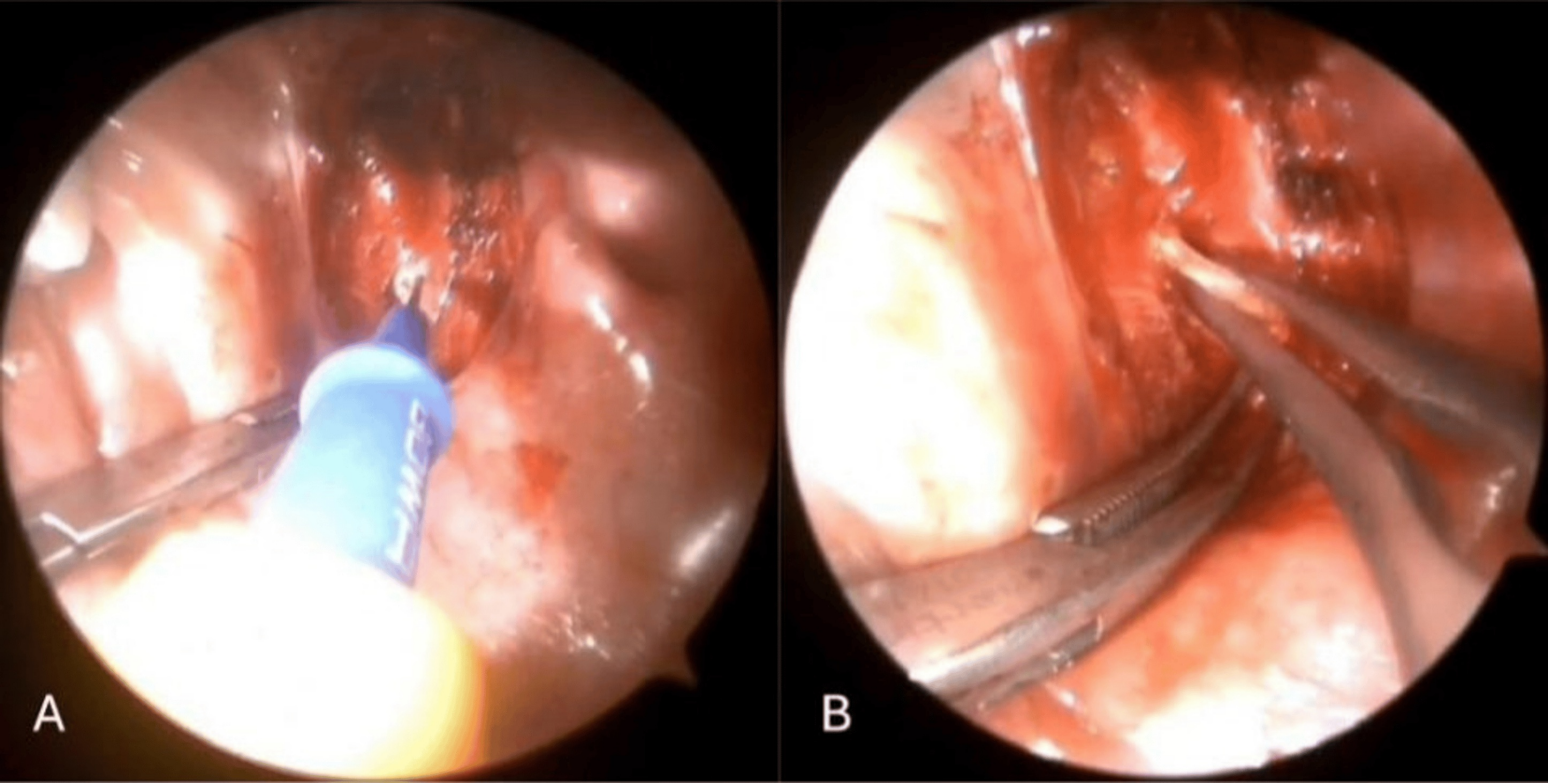
Skeletonization of the styloid process via dissection of its supporting structures (A). Transection of the styloid process using Luer bone-cutting forceps (B).

**Figure 4:**
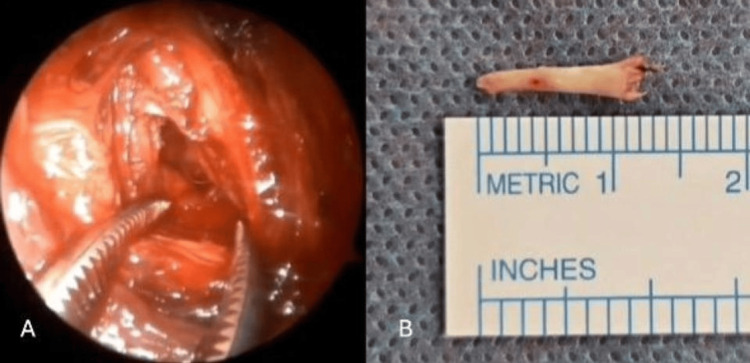
Evaluation of the resection bed of the styloid process (A). Excised segment of the styloid process (B).

The superior pharyngeal constrictor muscle was then dissected at the level of the styloid process. The process was exposed beneath the buccopharyngeal fascia using Pean forceps (Figure 2B). Direct visualization of the process beneath the buccopharyngeal fascia minimized the risk of neurovascular complications during subsequent dissection. The process was then skeletonized from surrounding anatomical structures, including the stylohyoid ligament, stylohyoid muscle, stylomandibular ligament, and styloglossus muscle, using a monopolar cautery (Figure 3A). The process was then transected using Luer bone-cutting forceps (Figure 3B), and residual soft tissue attachments were trimmed using monopolar cautery. The resection bed of the styloid process was then carefully evaluated (Figure 4A). In this case, no major vascular structures, including the internal and external carotid arteries and the internal jugular vein, were visualized in close proximity to the resection bed. The lateral pharyngeal wall was then closed with Vicryl 3-0 absorbable sutures. Styloidectomy was first performed on the right side, followed by the left. The procedure on the left side was recorded using an endoscope operated by a scrub nurse (Video 1). The procedure was completed in 1 hour and 21 minutes, including the time required for intraoperative video setup. The excised segments of the styloid processes measured 12 mm on the left side (Figure 4B) and 9.5 mm on the right side. 

**Video 1:** Intraoperative Footage.

**Figure 5:**
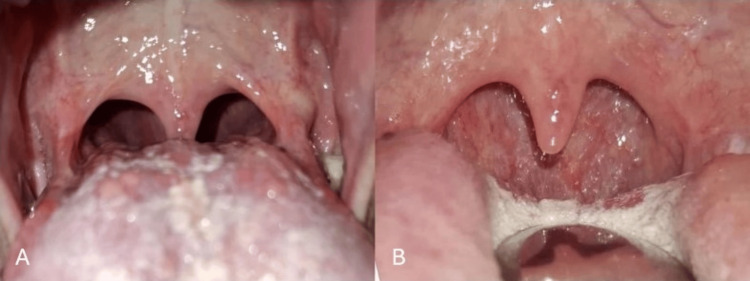
Photographs of the oropharynx and oral cavity taken during a follow-up visit after 5 months. Intraoral view of the oropharynx (A), and view following compression of the tongue base (B).

Postoperative treatment included anti-edema dexamethasone, administered once on the first postoperative day; prophylactic antibiotic therapy with amoxicillin and clavulanic acid for seven days, and as-needed analgesic treatment (nimesulide, metamizole, and paracetamol), continued for up to 14 days. At the follow-up visit on postoperative day 6, a fibrous deposit was observed in the tonsillar fossa, which had partially resolved by day 13 after surgery. The overall healing process was uneventful. A follow-up evaluation conducted two months after surgery revealed complete resolution of symptoms on the right side and significant improvement on the left. At that point, mild tenderness at the base of the tongue persisted on the left side but had resolved by the five-month follow-up visit. Photographs of the oropharynx and oral cavity taken during a visit after 5 months are shown in Figure 5. At that visit, an additional clinical examination of cranial nerves VII, IX, X, XI, and XII confirmed normal function. Postoperative pain was retrospectively assessed at the five-month follow-up visit using the Visual Analog Scale (VAS, 0-10). The patient recalled a pain level of 7 during the initial postoperative days, 3 at two weeks, 1 after two months, and 0 at five months.

## Discussion

Despite the substantial body of scientific literature on Eagle syndrome and the surgical techniques used in its management, no surgical gold standard has been established to date ​[9]​. The two most commonly used approaches are transoral styloidectomy and transcervical styloidectomy ​[9,15]​. Each technique offers distinct advantages and limitations, and the existing literature does not provide a clear consensus on the superiority of one approach over the other ​[2,9]​. 

When the elongated styloid process is in close proximity to the palatoglossal arch or the palatine tonsil, its terminal segment can often be palpated during a physical examination of the oral cavity and pharynx. This anatomical relationship enables the styloidectomy procedure to be readily performed via a transoral approach ​[15]​. During the procedure, palpation-based identification of the styloid process eliminates the need to search for it in the parapharyngeal space, thereby reducing the risk of complications and shortening the operative time. Once the styloid process is identified and the fibers of the superior pharyngeal constrictor muscle lying over it are dissected, the process becomes visible directly beneath the buccopharyngeal fascia. This facilitates its removal with minimal risk of neurovascular injury. The reduced risk of nerve damage associated with the transoral approach primarily concerns the facial nerve, which runs lateral to the styloid process and lies outside the surgical field. Although the transoral approach requires experience, it is considered relatively straightforward for surgeons familiar with the anatomy of this region.

This approach is chosen not only by ENT specialists but also by maxillofacial surgeons due to its numerous advantages ​[10]​. The transoral approach is associated with a shorter operative time ​[10,16]​, absence of visible skin scarring, minimal risk of facial nerve branch paralysis, shorter hospital stay, lower procedure-related costs, and a less technically demanding procedure ​[9,17]​. 

Despite the aforementioned advantages, the transoral approach has several limitations, including a restricted surgical field and limited visual control. Anatomical factors such as a bulky tongue base, short neck, limited mouth opening, or restricted neck extension, which typically complicate tonsillectomy, can also hinder the feasibility of transoral styloidectomy. Additionally, this approach may be associated with an increased risk of deep cervical infection. Notably, in some cases, partial resection of the styloid process via the transoral approach may be insufficient to alleviate symptoms, necessitating reoperation through a transcervical approach ​[1,9,18]​. Postoperative pain during swallowing and speaking, attributed to the associated tonsillectomy, is also reported more frequently following transoral procedures ​[15]​. A case of transient trismus has been described in the literature as a complication specific to this approach ​[9]​. 

A review study analyzing a relatively large number of cases (N=112) showed that the complication rate for the transoral approach (n=48) was 4.3%, notably lower than 16.3% reported for the transcervical approach (n = 64) ​[9]​. Importantly, no major complications associated with transoral styloidectomy, such as hemorrhage, infection, or nerve injury, were observed, supporting the relatively high safety profile of this technique. 

The limitations of the transoral approach can be partially addressed through appropriate modifications. The use of an endoscope or robotic assistance provides enhanced visualization of the limited surgical field, which, among other benefits, facilitates the management of potential intraoperative hemorrhage ​[10,11,18]​. Perioperative and postoperative antibiotic therapy helps reduce the risk of deep neck infections. Performing styloidectomy without tonsillectomy has been reported to reduce postoperative pain ​[19,20]​. 

## Conclusions

Transoral styloidectomy is considered a relatively safe surgical procedure, especially when the styloid process is palpable along the lateral pharyngeal wall. Compared to the transcervical approach, it has a lower risk of complications, particularly in terms of nerve injury. In addition, it is associated with superior cosmetic outcomes and a shorter operative time, although it carries a risk of postoperative complications similar to those of a tonsillectomy. The visual documentation presented in this report provides a detailed overview of this procedure and serves as an effective educational tool, facilitating the training of surgeons in this relatively straightforward but rarely performed technique. 
